# *Alexandrium* spp.: From Toxicity to Potential Biotechnological Benefits

**DOI:** 10.3390/md22010031

**Published:** 2023-12-30

**Authors:** Eleonora Montuori, Daniele De Luca, Antonella Penna, Darta Stalberga, Chiara Lauritano

**Affiliations:** 1 Department of Chemical, Biological, Pharmaceutical and Environmental Sciences, University of Messina, Viale F. Stagno d’Alcontres 31, 98166 Messina, Italy; eleonora.montuori@studenti.unime.it; 2 Department of Ecosustainable Marine Biotechnology, Stazione Zoologica Anton Dohrn, Via Acton 55, 80133 Napoli, Italy; 3 Research Infrastructure for Marine Biological Resources Department, Stazione Zoologica Anton Dohrn, Villa Comunale, 80121 Napoli, Italy; daniele.deluca@szn.it; 4 Department of Biomolecular Sciences, University of Urbino, Campus E. Mattei, 61029 Urbino, Italy; antonella.penna@uniurb.it; 5 Department of Biomedical and Clinical Sciences, Division of Clinical Chemistry and Pharmacology, Linköping University, SE-58183 Linköping, Sweden; darta.stalberga@liu.se

**Keywords:** *Alexandrium* spp., toxins, harmful algal blooms, anticancer, biotechnological applications

## Abstract

Many dinoflagellates of the genus *Alexandrium* are well known for being responsible for harmful algal blooms (HABs), producing potent toxins that cause damages to other marine organisms, aquaculture, fishery, tourism, as well as induce human intoxications and even death after consumption of contaminated shellfish or fish. In this review, we summarize potential bioprospecting associated to the genus *Alexandrium*, including which *Alexandrium* spp. produce metabolites with anticancer, antimicrobial, antiviral, as well as anti-Alzheimer applications. When available, we report their mechanisms of action and targets. We also discuss recent progress on the identification of secondary metabolites with biological properties favorable to human health and aquaculture. Altogether, this information highlights the importance of studying which culturing conditions induce the activation of enzymatic pathways responsible for the synthesis of bioactive metabolites. It also suggests considering and comparing clones collected in different locations for toxin monitoring and marine bioprospecting. This review can be of interest not only for the scientific community, but also for the entire population and industries.

## 1. Introduction

Dinoflagellates represent one of the most important groups of marine phytoplankton, with more than 1500 species in the marine environment [1] and a total of around 2400 species also considering freshwater habitats [2,3]. This high species richness is also reflected when encompassing diverse biochemical (pigments and toxins produced), metabolic (autotrophs, mixotrophs, and grazers) and ecological (free-living, symbionts, and parasitic) strategies [4,5,6,7]. Some species have been the object of in-depth investigations due to their importance in ecosystem stability and resilience (e.g., zooxanthellae in coral reefs) or production of toxins with relevant impact on human health and activities (e.g., aquaculture). Regarding the latter aspect, some dinoflagellate species are responsible for harmful algal blooms (HABs), producing potent toxins that cause human intoxications and even death after consumption of contaminated shellfish or fish [8]. Moreso, these toxins can cause damage to aquaculture and the fishery industry [9,10], impairment of tourism and recreational activities [11,12], alterations in marine trophic structures [13,14], and mortality events of marine mammals, fish, and birds [15,16]. The globally distributed genus *Alexandrium* Halim contains 33 accepted species [17], of which some are involved in the formation of HABs (algal grow out of control which have toxic or harmful effects on humans or marine organisms and may differ in terms of severity, diversity, and distribution [18,19,20]). As a consequence, this genus is well studied among dinoflagellates [20]. The taxonomy of dinoflagellates, thanks to morphological and molecular analyses, is continuously improving with occasional controversies. For instance, John et al. 2014 [21] assigned the *Alexandrium* species names as Group I, *A. fundyense*; Group II, *A. mediterraneum*; Group III, *A. tamarense*; Group IV, *A. pacificum*; and Group V, *A. australiense*.

Previous reviews on *Alexandrium* are available: Long et al. in 2021 focused on *Alexandrium* bioactive extracellular compounds (BECs) [22], Lewis et al. in 2018 reviewed global distribution of *Alexandrium minutum* with a focus on Northern Europe [23], Casabianca et al. in 2012 investigated the population genetic structure and connectivity of *A. minutum* in the Mediterranean Sea [24], while Bi et al. in 2019 focused on studies in dinoflagellates, summarizing *Alexandrium* papers on expressed sequence tags, transcriptomes and other molecular approaches [25]. Our review is the first to discuss *Alexandrium* potential also for marine bioprospecting. Considering the harmfulness of many *Alexandrium* species, several studies have focused on the identification of toxins, enzymatic pathways responsible of their synthesis [26], transcriptomics [26,27,28], metabolomics [29], as well as various detection methods, ranging from microscopic examinations to real-time PCR-based assays [30,31]. However, species which do not produce toxins have been shown to exert bioactivities [32]. Our review highlights which *Alexandrium* produces metabolites with possible biotechnological applications, such as with anticancer, antimicrobial, antiviral, as well as anti-Alzheimer activity. This review explores their mechanisms of action and targets, where such information is available.

**Figure 1:**
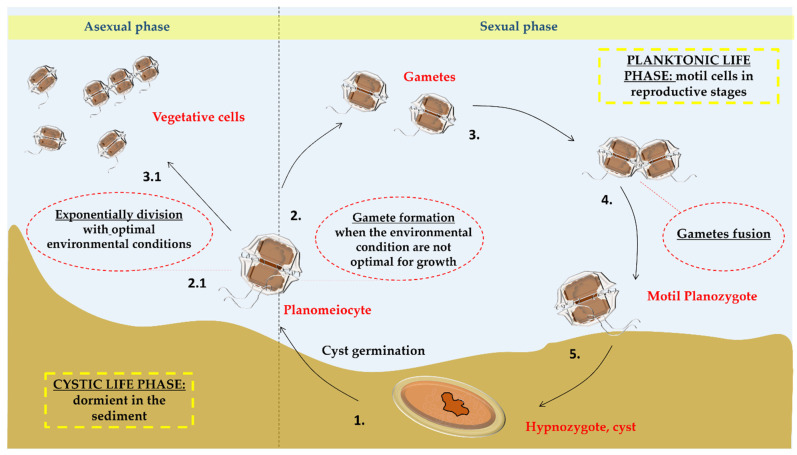
Life cycle of *Alexandrium* spp. The life cycle of *Alexandrium* spp. is divided into two phases, the planktonic vegetative phase with asexual reproduction and the dormancy phase in the sediment in the form of cysts resulting in sexual reproduction. The duration of each phase is influenced by the conditions in the surrounding environment (temperature, pH, nutrients, etc.). The cycle starts from the germination of the cyst in planomeiocyte (1.) that under favorable conditions for growth undertakes asexual reproduction by demoschisis giving rise to the vegetative phase of the cells (2.1; 3.1). Under not optimal conditions of growth, it gives rise to the sexual reproduction forming two haploid gametes (2.) that subsequently merge (3.), forming the diploid and biflagellated planozygote (4.) which forms a new cyst called hypnozygote (5.).

*Alexandrium* cells are roughly spherical. They occur as solitary or in short chains in rapidly growing populations, and possess two flagella that allow vertical migrations in the water column [33]. The genus includes both autotrophic and mixotrophic species (see Lim et al. [34] and references therein), with the latter nutritional strategy triggered by specific life phases and conditions [18]; bioluminescence is also reported for some species [24]. Like other dinoflagellates, some *Alexandrium* species possess a two-phase life strategy (Figure 1): a planktonic phase characterized by motile cells at different reproductive stages, and a cystic life phase where cells lie dormant in the sediments [35,36]. The cyst phase is an adaptation that allows survival during unfavorable growing conditions and is at the basis of the formation of blooms when favorable conditions for life return. Such blooms occur seasonally and are influenced by abiotic (temperature, salinity, nutrient availability) and biotic (predation, viral infection) factors [36].

As other microalgae, *Alexandrium* species produce primary and secondary metabolites, but, among these, they may produce toxic substances [20,37]. Toxic *Alexandrium* species are summarized in Table 1. Saxitoxin (STX), a tetrahydropurine alkaloid also known as paralytic shellfish toxin (PST), is one of the toxins produced by the genus *Alexandrium* and it is known to be one of the most toxic and harmful substances to humans. Saxitoxin was isolated and characterized in 1966 by Schantz et al. from *Alexandrium catenella* [20,38]. The lethal dose corresponds to 1–4 mg/person, depending on the sex and physiological predisposition of the individual [18,39,40]. 

Saxitoxin is also known for its use as a chemical agent in war [67]. It is also present in the Schedule I annex list of toxic chemicals of the Chemical Weapons Convention available on the Organization for the Prohibition of Chemical Weapons Chemicals website (https://www.opcw.org/chemical-weapons-convention/annexes/annex-chemicals/schedule-1; accessed on 21 October 2023) [67] and this is a further confirmation of its dangerousness and potential use as a weapon.

**Figure 2:**
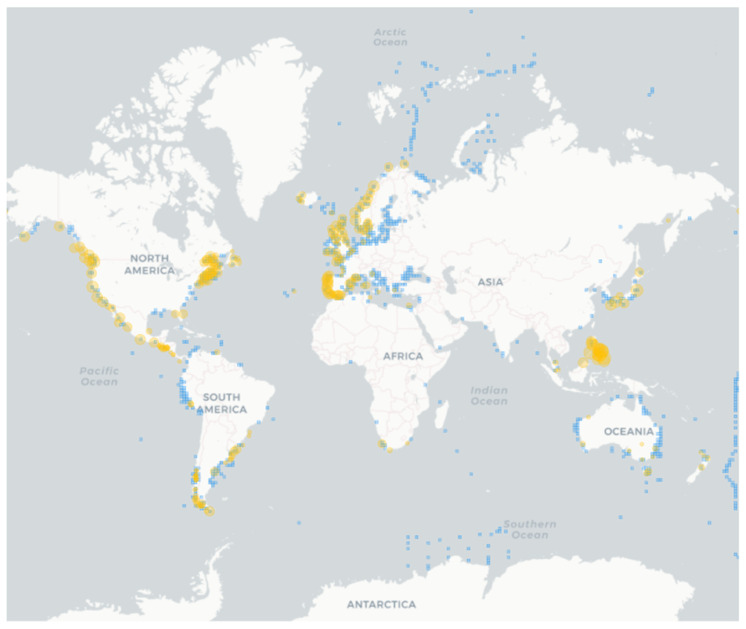
Occurrence of *Alexandrium* spp. (light blue squares) and paralytic shellfish poisoning (PSP) events (orange). Species occurrences are from 1904, while PSP records from 1938. The map was built using the Harmful Algal Information System (HAIS) available at https://data.hais.ioc-unesco.org/ (accessed on 11 August 2023).

The first record of paralytic shellfish poisoning (PSP) events dated back to the end of 18th century in British Columbia [68], but regular data on such events were available from the end of 1930′s. Evidence showed that the phenomenon was reported along the coasts worldwide and mostly matched the known distribution of *Alexandrium* species (Figure 2). The genus *Alexandrium* produces other bioactive lipophilic compounds, called phycotoxins, such as spirolides, gymnodimines, goniodomins. The only known species that produces spirolides is *A. ostenfeldii* which also produces, gymnodimines and goniodomins [69,70]. The macrocyclic polyketide Goniodomin was produced also by *A. hiranoi* [71], *A. monilatum* [53], *A. pseudogonyaulax* [61], and *A. taylorii* [22,66].

## 2. *Alexandrium* Secondary Metabolites and Possible Biotechnological Applications

**Figure 3:**
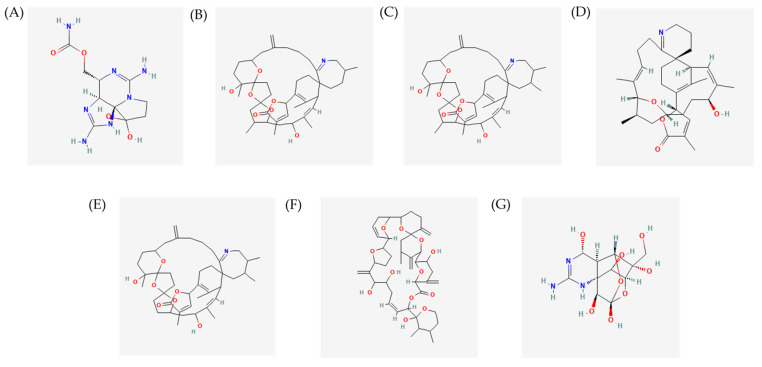
Chemical structures of: (**A**) Saxitoxin [72]; (**B**) Spirolide A [73]; (**C**) Spirolide C [74]; (**D**) Gymnodimine A [75]; (**E**) 13-desmeethyl spirolide C [76]; (**F**) Goniodomin A [77]; (**G**) tetrodoxin [78].

As mentioned in the previous paragraph, *Alexandrium* spp. can produce large quantities of varieties of molecules, including toxins such as saxitoxin, cyclic imine toxins, goniodomines and tetrodotoxin. Each toxin may have a similar structural variant, and each analogue may have different levels of toxicity and biological activity. Therefore, it is essential to continue the search and characterization of new analogues of these toxins for the assessment of their toxic effects and possible food safety measures to be taken into account for the prevention of intoxication by their ingestion. We report below the characteristics of the main toxins actually known and summarize the *Alexandrium* compound and extract bioactivities. Figure 3 summarizes the available chemical structures of *Alexandrium* compounds reported to exert possible bioactivities useful for human applications.

### 2.1. Saxitoxin 

#### 2.1.1. Chemistry

Saxitoxin (STX) is a compound belonging to the alkaloid class with the molecular formula C_10_H_17_N_7_O_4_ (Molecular weight = 299) and is composed of a 3,4-propinoperhydropurine tricyclic system (Figure 3A). Saxitoxin was the first PST whose purification dates back to 1957. Since then, 57 analogues of saxitoxin have been described [67,79]. The presence of two guanidinium groups in its structures makes this molecule highly polar [38,80]. Saxitoxin and its structural analogues differ in the fractions of the lateral groups (R_1_–R_4_) and, therefore, are commonly grouped according to these variable residues. They can be structurally classified as non-sulfated, such as STX and neosaxitoxin (neoSTX), mono-sulfated, such as gonyautoxins (GTXs 1–6), or di-sulfates (C1–4 toxins), decarbamoylated as decarbamoyl-saxitoxins (dcSTX, dcneoSTX), and deoxy-decarbamoylated like decarbamoyl-gonyautoxins (dcGTXs 1–4), and the 13-deoxy-decarbamoyl derivatives (doSTX, doGTX 2,3) [40,79,81]. 

#### 2.1.2. Toxicity

Given the structural variation between saxitoxin and analogues, there is also a fluctuation in the toxicity range of these PSTs which varies by two orders of magnitude ranging from 15 MU·μmol^−1^ of C1 toxin to 2483 MU·μmol^−1^ of STX [81]. The proton group 7,8,9 guanidinium has been shown to be the key site to the bioactivity of the toxin as it allows efficient binding to Na [40]. It blocks the ion flux by binding the resting receptor site of the channels NaV1.1.–NaV1.4 and NaV1.6, which are the one of 10 subtypes of the alpha subunits of the NaV channels [82,83,84]. Saxitoxin acts by interacting with the extracellular regions of the Nav channels, without any dependence on tension [83]. However, it also binds to calcium [85] and potassium channels [86]. Saxitoxin is not directly involved in blocking both channels as in the case of NaVs. In K+ channels, 4 saxitoxins molecules bind to the extracellular site causing strong transmembrane depolarization that causes the canal to open and reduce potassium exchange [67,86]. In calcium channels, saxitoxin is associated with binding to the selectivity filter, but this binding does not cause complete blockage of the channel [85,87]. Llewellyn et al. in 2008 also observed that saxitoxin behaved as a weak inhibitor of nitric oxide neuronal synthase, which catabolizes the production of nitric oxide from the guanidine group of arginine [40]. To date, there is no clinically approved antidote on the market for poisoning with saxitoxin or its derivatives. The only common treatment is with activated carbon and application of artificial respiration in the early stages of poisoning [88]. 

#### 2.1.3. Possible Applications

Toxins produced by the genus *Alexandrium* play an important role in research. For instance, saxitoxin and its analogues have been used to study and understand how sodium potassium channels work [22].

In 2000, saxitoxin, neosaxitoxin, decarbamoyl saxitoxin, and tetrodotoxin were tested for rat sciatic nerve block, exploring their potential as agents for extending the duration of local anesthesia [89]. Lethality, onset, and duration of action for thermal analgesia (hot-plate testing) and motor block (weight-bearing) were evaluated. Analgesia for 60 min was obtained with neosaxitoxin at 34 ± 2 micromol/L, saxitoxin at 58 ± 3 micromol/L, TTX at 92 ± 5 micromol/L, and decarbamoyl saxitoxin at 268 ± 8 micromol/L [89]. Wand et al. in 2006 detected the presence of goyautoxins in ATGX02 strain of *Alexandrium tamarense* isolated near Weizhou Island in the Beibu Gulf [81], while Xu et al. in 2021 found that *Alexandrium tamiyavanichii* isolated from Beibu Gulf waters in China produces low levels of saxitoxins and gonyautoxins [90]. The gonyautoxins are phycotoxins that act by reversibly blocking voltage-gated sodium channels at axonal levels. In Lettes et al. 2009, the therapeutic properties of gonyautoxin 2/3 epimers local infiltration in chronic tension-type headache patients were evaluated. The clinical trial was conducted from September 2004 to 2005 in Hospital Clínico Universidad de Chile. Twenty-seven patients with chronic tension-type headaches aged 18–80 years old were locally infiltrated with a dose of 50 µg of gonyautoxins. A total of 70% of patients responded to the treatment with long-lasting effects [91]. 

### 2.2. Spiroimines (Gymnodimines and Spirolides)

#### 2.2.1. Chemistry

Both gymnodimines and spirolides are members of the spiroimine group of lipophilic macrocyclic imine toxins [92]. Together with prorocentrolides, spiro-prorocentrimine, pinnatoxins, and pteriatoxins, they form a part of the cyclic imine (CI) family and are collectively linked to sporomine mollusc poisoning (SMP) [93,94]. The CI family toxins are characterized by macrocyclic compounds with imine, i.e., a double carbon–nitrogen bond, and spiro-linked ether moieties [95]. The spiroimine subunit has a substantial carbocyclic polyether structure, ranging from 14 members for portimines to 27 members for pinnatoxines. Spirolides and gymnodimines have a carbocycle of 23 and 16 members, respectively [96,97]. Currently, 17 individual members of the spirolides subgroup have been identified [98]. An important feature to consider regarding spirolides is their water stability, as it is parallel to the potency of these marine toxins. Spirolides A (Figure 3B) and B are known to be unstable, while a C32-monomethyl fraction is rapidly hydrolyzed to more stable and common spirolides such as E and F. Spirolides C (Figure 3C), D, G, H, and I instead have an imine ring replaced with 31,32-dimethyl and appear to be much more resistant to hydrolysis [96].

On the contrary of spirolides, gymnodimines have been detected in smaller amounts and variety in microalgae [98]. To date, eight members of gymnodimines have been described [98]. Gymnodimines are the smallest members of the cyclic imine family by molecular weight (with a molecular weight of 507.7 g/mol) [96]. Gymnodimines are characterized by a labile 6,6-spirocyclic imine fragment embedded in a 16-membered carbocycle incorporating a tetrahydrofuran ring and one (GYM (A))5 or two (GYMs B11 and C)12 trisubstituted E-double bonds [96]. 

#### 2.2.2. Toxicity

Generally, CI toxins are antagonists of muscle and neural-type nicotinic acetylcholine receptors (nAChRs) [99]. Specifically, Gymnodimine A (Figure 3D) and 13-desmethyl spirolide C (Figure 3E) express neurotoxicity by broadly targeting and reversibly blocking with high-affinity nicotinic acetylcholine receptors (nAChR) [100,101]. The toxicity of CId as “fast acting” induces a set of neurological symptoms followed by death in mouse bioassays [102]. Other observed negative impacts of the CI toxins are expressed on neuromuscular, sensory, digestive, and respiratory systems [93,94]. Munday et al. in 2004 [102] observed that the gymnodimine expressed high toxicity in mouse by injection (LD_50_ = 96 µg/kg) However, when gymnodimine was ingested together with food, exceeding the LD_50_ approximately 10 times of oral LD_50_, it expressed low toxicity [102]. Spirolides express a similar toxicity profile of decreased toxicity when the toxin is ingested with food [103]. In 2001, three new spirolides derived from *Alexandrium ostenfeldii* called Spirolide A, Spirolide C, and 13-Desmethyl spirolide C were isolated [104].

#### 2.2.3. Possible Applications

The compound 13-Desmethyl spirolide C is of strong pharmacological interest as it targets nicotinic receptors. In 2006 [105], in vivo studies were conducted in mice 3xTg-AD affected by Alzheimer’s, and 11.9 µg/kg of 13-desmethyl spirolide C by intraperitoneal injections was given. Authors noted that 2 min after injection, 13-desmethyl spirolide C was located in the brain and remains detectable even 24 h after administration. The 13-desmethyl spirolide C induced in mice 3xTg-AD positive effects on Alzheimer’s disease markers with an increase in N-acetyl aspartate (NAA) levels, an increase in synaptophytin, and a decrease in intracellular amyloid beta in the hypothalamus, further concluding that the 13-desmethyl spirolide C was able to cross the blood–brain barrier, making this a toxin of inspiration for the development of new drugs for neurodegenerative diseases [105].

### 2.3. Goniodomins

#### 2.3.1. Chemistry

Goniodomins are characterized as polyether macrolide compounds consisting of 32- membered macrolactone with an array of five- and six-membered cyclic ethers [106]. 

#### 2.3.2. Toxicity

These toxins have lipophilic characteristics and are stored intracellularly and expressing significantly higher toxicity when released in the environment upon cytolysis [53,106]. However, even slight changes in the chemical structure of Goniodomin A (Figure 3F) induces a large change in its cytotoxicity. Goniodomin A, however, expresses a toxicity 10 times higher than Goniodomin B. For the latter, the opening of the C-ring and the isomeric changes between C-24 and C-29 appear to be the modifications responsible for a decrease in the toxicity of the molecule [49].

#### 2.3.3. Possible Applications

The Goniodomin A toxin has a wide range of biological activities, such as the inhibition of fungal growth, the termination of cell division in fertilized sea-urchin eggs, and even causes morphological changes in the liver [49,106]. Goniodomins also disturbs the actomyosin ATPase activity by binding stoichiometrically to actin monomers in filaments, causing conformational changes. This activity is highly affected by myosin and the toxin disturbs the ATPase activity if its concentration is >10^−6^ M, otherwise the ATPase activity is enhanced [49,107].

Some suppressive angiogenic activity in endothelial cells induced by Goniodomin A has also been found acting by inhibition of actin reorganization. The presence of Goniodomin confirmed this property by inhibiting angiogenesis in vivo. It also inhibited the development of the vascular system in a chorioallantoic membrane, but no effect was observed on the already established structures of the strain fiber formed with G-betagamma expression (Gβγ) [108].

Goniodomin A is also believed to express neurotoxicity. It induced an increase in cytosolic calcium and depolarization of the cell membrane when this ion is present in the extracellular medium. In addition, as in the study by Espiña et al. in 2016, neuroblastoma cells were more sensitive to the toxin than rat hepatocytes when they affected their metabolic rate, concluding that Goniodomin A may induce high neurotoxicity if it reaches the nervous system [49].

Mizuno et al. in 1998 reported that Goniodomin A induced morphological changes in human brain astrocytoma cells by increasing the filamentous actin content. The cells were becoming refractile from flat and it was accompanied by the outgrowth of a needle-like structures from the cell surface [109]. Successively, Matsunaga et al. confirmed that even if Goniodomin A was significantly inhibited by the troponin–tropomyosin complex, as it induced a change in the distribution in the same human astrocyte cell line (1321N1 cells) by increasing the intracellular content of F-actin [107]. The authors also demonstrated that Goniodomin A inhibited ATPase activities of atrial myofibrils, natural actomyosin, and reconstituted actomyosin [107]. Goniodomin A is known for its ability to suppress the angiogenic properties of endothelial cells at least in part through the inhibition of actin reorganization in cells [108].

### 2.4. Tetrodotoxin

#### 2.4.1. Chemistry

Tetrodotoxin (TTX; Figure 3G), also called heterocyclic organic molecule hydroperquinozolinine (aminoperhydroquinazo-lone), is an orthoester with a group of guanidinium and a system of polyoxygenated rings, similar to saxitoxin, found in *Alexandrium tamarense* [110,111]. This toxin is heat stable, water soluble [112], and degrades at alkaline pH’s [110]. The TTX structure is similar to a prism with a tetrahedral core [110]. There have been 30 structural analogues of the tetrodotoxin reported, of which 26 are known to be naturally occurring [113,114].

#### 2.4.2. Toxicity

The active moiety of tetrodotoxin, same as for saxitoxin, is known to be the only 1,2,3 guanidinium moiety. It is connected to a highly oxygenated carbon skeleton that possesses a 2,4-dioxaadamantane portion containing five hydroxyl groups and expresses its bioactivity when the guanidium group is protonated [110,115]. The toxicity degree of tetrodotoxin is also affected by the number and position of hydroxyl groups present in the structure. As demonstrated by Yang and Kao reported in 1998, hydroxyl groups at C-4, C-6, C-9, C-10 and C-11 are involved in the binding of the sodium channel [116]. Subsequently, Yotsu-Yamashita et al. in 1999 confirmed the involvement of hydroxyl groups to tetrodotoxin C-6 and C-11 in acting as hydrogen bond donors for sodium channel binding [113]. 

The guanidinium moieties on tetrodotoxin and on its analogs are the pharmacophores, and therefore are responsible for the blocking activity on the NaV channels [82]. The binding of tetrodotoxin to NaV results from the interaction between the positively charged guanidine group on the TTX with the negatively charged carboxylate groups on the side chains in the mouth of site 1 of the sodium channel, thereby partially or completely blocking the inward Na^+^ ion current [117,118].

The guanidinium toxins express toxicity to various ion channel types, but their primary target is voltage-gated ion channel blockage by binding to the NaV channels of excitable cells, mainly in nerve and muscle cell [82,110]. Tetrodotoxin binds and inhibits specifically six out of nine sodium channel isoforms expressed in mammalian cells: NaV1.1, NaV1.2, NaV1.3, and NaV1.7 [82,119,120]. It then terminates the nerve conduction and the muscle action potentials [121] leading to progressive paralysis and, in extreme cases, to death because of respiratory and heart failure [122,123]. Its toxicity is often emphasized by comparing it as being over a thousand times more toxic to humans than cyanide [124]. Even now, tetrodotoxin has no known antidote which is officially approved [125]. The only treatment for the toxin in case of intoxication is observation and the appropriate supportive care [124]. The supportive care includes practices such as gastric lavage with activated charcoal to remove unabsorbed toxin and machine-assisted ventilation when respiration is severely affected in conjunction with the use of dopamine and atropine to correct a problem with hypotension and arrhythmia, accordingly [126,127,128]. Typically, symptoms are expressed within 1 to 6 h if the toxin is ingested via fish [125].

#### 2.4.3. Possible Applications

As summarized by Nieto et al. in 2012 [129], the administration of tetrodotoxin has been evaluated in both humans and experimental animals under different pain conditions [129]. In Hagen et al. 2017, the bioactivity of tetrodotoxin was investigated by daily subcutaneous injection for four consecutive days of 30 µg of tetrodotoxins in patients randomized to receive tetrodotoxins or placebo. All the patients were diagnosed with cancer. A clinically important analgesic signal was shown to moderate pain related to the tetrodotoxin cancer group [130]. An example of a drug based on tetrodotoxin is Halneuron^®^, a pain medication drug undergoing phase 3 clinical trials as a new class of non-opioid analgesics (https://wexpharma.com/; accessed on 27 December 2023). This drug was developed for the treatment of moderate-to-severe chemotherapy-induced neuropathic pain, cancer pain, and other pain indications. It has been already tested on 700 people in over 15 clinical trials and is going for a double-blinded, placebo-controlled Phase 3 trial (https://wexpharma.com/; accessed on 27 December 2023).

### 2.5. Other Molecules 

Galasso et al. in 2018 [131] investigated the anti-cancer activity of *Alexandrium minutum* against a panel of cancer cell lines such as human pancreatic cancer cells PC-3, human colorectal adenocarcinoma cells HT29, and human lung adenocarcinoma cells A549. The authors found a specific anti-proliferative fraction from the total extract of *A. minutum* against A459, named fraction 3B. The activity was specific against A549 with an IC50 of 0.4 µg/mL. They evaluated the activation of the mitophagy pathway with a differential expression gene analysis. After a cell treatment with 0.4 µg/mL of fraction 3B of *A. minutum* for 2 h, it was observed that a strong up-regulation of the autophagy-related protein 12/gene (Atg12) with a consequent increase in ATPase H+ Transporting V1 Subunit G2 (ATP6V1G2) and BCL2/Adenovirus E1B19 kDa Interacting Protein 3 (BNIP3) occurred. In addition, there was up-regulation of two genes involved in mitophagy: PTEN induced putative kinase1 (PINK1) and the Parkin gene. The authors did not find the same cytotoxic and mitophagy activities against the normal lung fibroblast WI38, used as a control cell line. After a chemical analysis by colorimetric the phenol–sulphuric acid method and Breadford assay, Galasso et al. in 2018 revealed that this fraction was rich in carbohydrates (94%) and protein (4%) [131].

Yamasaki et al. 2008 [132] isolated a polysaccharide-based toxin from the dinoflagellate *Alexandrium tamarense*. They purified the AT-toxin by gel filtration on the fast protein liquid chromatography-FPLC system and thanks to sugar composition assay, noted that this toxin contains galactose, fucose, mannose, N-acetylglucosamine, xylose, and other minor saccharides. The polysaccharide-based AT-toxin showed cytotoxic effects against different cell lines such as the HeLa human immortalized cervix carcinoma cells, Vero human kidney epithelial cells, XC rat Rous Sarcoma cells, CHO Chinese hamster ovary epithelial cells, and U937 human monocyte Histiocytic Lymphoma cells. On U937, the treatment with polysaccharide-based TA-toxin induced a consistent cytotoxicity with an IC50 of 110 ng/mL. The authors demonstrated that AT-toxin was an apoptotic inducible toxin by measuring the increasing DNA fragmentation that is a hallmark of apoptotic cell death in U937 cells by diphenylamine assay after treatment with TA-toxin [132].

Sansone et al. 2018 [133] also found that the *Alexandrium andersoni* raw methanol total extract and the solid-phase extraction-SPE fractions induced high cytotoxicity on HT29 human colorectal cancer cells and A549 human lung adenocarcinoma cells, while they did not observe cytotoxicity for the two normal fibroblast cell lines WI38 and BEAS-2B. Fraction B was the most active on A549 cells, with the minimum concentration for inhibition cell viability ≤ 1 µg/mL, while fraction D was the most active fraction on HT29 cells, where the minimum concentration for inhibition of cell viability was >1 μg/mL. Nuclear magnetic resonance-NMR and liquid chromatography-mass spectrometry LC-MS spectra supported a different distribution of the metabolites in SPE fractions. The glycolipids, mostly digalactosyl diacyl glycerol (MGDG) and sulfoquinovosyl diacyl glycerol (SQDG), were the predominant metabolites in fraction B, whereas fraction D was mostly composed by sterols (ST) and triacylglycerols (TAG). Sansone et al. found that *A. andersoni* activated two different cell death pathways in the two tumor cell lines. Both signaling pathways observed were triggered by tumor necrosis factor receptor (TNFR), as observed by a gene expression analysis after 2 h of treatment with 1 μg/mL concentration of the two fractions in A549 and HT29. They noted that SPE fraction B was able to induce a down-regulation of anti-apoptotic molecules (BIRC3 and TRAF2) and an up-regulation of death receptors (CD27, DR3, TNF, and TNFS8) in A549 cells. In HT29 cells, it was observed that fraction D directly induced cell death through a particular TNF-related pathway also involving DNA cell-signaling damage. Two tumor necrosis factors, FASLG and TNF, were over-expressed as well as Gadd45 alpha and gamma, and Foxl1, confirming a cell-signaling pathway in response to the DNA damage. Gadd45 alpha and gamma proteins play important roles in suppressing cell proliferation, mediating cell cycle arrest, promoting apoptosis, inducing DNA repair, and stabilizing chromatin assessment [116].

De vera et al. in 2018 [134] conducted a bioprospecting study on the extracts of 33 strains of microalgae. Between these strains, they found that the extracts of the microalga *Alexandrium tamarense* had an anti-proliferative activity at concentration of 50 µg/mL against tumorigenic breast cancer cell MCF-7, non-tumorigenic breast cancer cell MCF-10A, and the two tumorigenic cell lines of prostate cancer cells LNCaP and PC-3. Authors also evaluated the apoptotic activity of *A. tamarense* extracts with an annexin V binding assay, showing that it induced apoptosis on the human hepatocellular carcinoma cell line HepG2 tested at 100 µg/mL. Raw extracts of *Alexandrium andersonii* also showed a weak cell death on human lung carcinoma A549 and on colorectal carcinoma HT29 cell lines [134]. Lauritano et al. in 2016 also evaluated the antiproliferative activity against human melanoma A2058 cells of 32 microalgal species, including three *Alexandrium*, i.e., *A. andersonii* (FE108), *A. tamutum* (FE107), and *A. minutum* (FE126) from the Mediterranean Sea [32]. In particular, they found that FE108 and FE107 reduced almost to 0% A2058 cell viability already at 10 µg/mL. FE107 extract (FE107/3) derived from a culture in phosphate starvation had weaker effect, showing antiproliferative activity at 25–100 µg/mL. Finally, FE126 reduced cell viability at 80% at 50 µg/mL, while at 100 µg/mL cell viability was reduced at 30%. All the three *Alexandium* species also showed cytotoxicity on normal human lung fibroblast (MRC-5). The authors showed how different culturing conditions as well as different clones of the same species may exert different bioactivities. The compounds responsible of those activities are still not characterized [32].

Some species of *Alexandrium* genus also produce a series of bioactive extracellular compounds (BECs) not yet completely characterized. One example of characterized BEC is alexandrolide, a microalgal growth inhibitor isolated from *A. catenella* which also showed cytotoxicity against mouse lymphoid P388. The production of BECs has an energy cost for the cell like the production of any organic molecule, but the mechanisms for their production are still not clear [22]. Extracellular particles produced by some species of *Alexandrium* may have anti-parasitic activities [22]. Long et al. [135] showed *Alexandrium minutum* activity against the dinoflagellate parasite *Amoebophrya* sp. The authors noted that exposure of free-life infectious spores of *Amoebophrya* sp. to the filtered exudates with allelochemical properties of *A. minutum* affected the mortality rate of *Amoebophrya* sp. In addition to the infectious capacity of the latter, exposure to *A. minutum* exudates quickly resulted in permeation of the membranes of *Amoebophrya* sp. dinospores, leading to the loss of dinofluorescence associated in turn with the loss in virulence of the parasite in cultures. After 20 min of exposure with *A. minutum* extracts, a reduction of 40% of viability was observed while after 2 h of exposure the reduction was almost 98% [135]. Lassudrie et al. in 2015 [136] observed the anti-viral properties of the species *Alexandrium catenella* against herpesvirus OsHV-1 μvar affecting oysters. When the herpes virus OsHV-1 μvar was exposed to *A. catenella*, the prevalence of viral infection in oysters decreased. The authors concluded that the reduction in viral infection is due to an antagonistic relationship that is established between the virus and toxic microalgae assuming various mechanisms of action. Firstly, there may be a direct interaction between OsHV-1 mvar and *A. catenella* that could reduce viral transmission and algae availability for oyster consumption, or the antagonistic action could be the consequence of immune and physiological responses to the virus and/or microalgae [136]. The potential direct deleterious effects of *Alexandrium* BECs on marine viruses can then mitigate the infection by decreasing the viral load. To our knowledge, the effects of BECs on pathogenic bacteria have not yet been studied. Bioactivities reported for *Alexandrium* spp. compounds and extracts are summarized in Table 2.

## 3. Conclusions

This review describes *Alexandrium* toxins and their possible useful applications. In particular, anti-cancer, anti-microbial, anti-parasite, and anti-Alzheimer activities have been reported for some *Alexandrium* compounds or extracts. These data highlight that even if some *Alexandrium* spp. are very toxic and dangerous for other marine organisms and humans, some strains may produce metabolites with possible beneficial effects for human health. This was also found for other toxic species such as cyanobacteria [138,139]. This testifies how important it is for biological inter-species diversity, and how it can protect and maintain this diversity for the health status of the marine environment and humans. The diverse range of compounds produced, both toxic and beneficial, can vary under different growth conditions, representing an opportunity for further research. By delving deeper into this aspect, it will be possible to identify growth conditions to enhance the production of specific compounds. By improving production efficiency and conducting in-depth molecular studies, we can gain a detailed understanding of the molecular and metabolic pathways involved in microalgae bioactive compound production. This knowledge can then be used to develop genome editing techniques that transform microalgae into a versatile biotechnological tool. One approach to achieve this is to increase transcriptomic studies on the same algae cultivated under various conditions (e.g., variation of temperature, pH, and salinity, as well as the composition of culture media) in order to identify differentially expressed genes involved in compound metabolism and potentially target them for silencing (e.g., by using RNA interference molecular techniques). This research approach holds great potential for enhancing the production of specific compounds and optimizing the beneficial properties of microalgae. Exciting research directions in light of bioactivity discovery useful for human health can be represented by the search for new *Alexandrium* strains/clones, new metabolites, and through evaluation of the growth conditions in which these compounds are mostly produced, as well as through considering the possibility of synthetic modification of some molecule structures to reduce side effects and increase specificity and efficacy. Results will be of interest not only for the scientific community, but also for human health and the pharmaceutical industries.

## Figures and Tables

**Table 1:** List of known toxic *Alexandrium* species. Species names are as in AlgaeBase [17] and only accepted names are shown.

Species	Toxins	Effects	References
*A. affine* (H. Inoue and Y. Fukuyo) Balech	Saxitoxins (only genes found)	Not evaluated	[41]
*A. andersonii* Balech *	Saxitoxins (GTX2, NEO, STX)	Inhibition of voltage-gated sodium channels, hemolytic activity	[42,43]
*A. catenella* (Whedon and Kofoid) Balech	Saxitoxins (GTX1, GTX4, NEO, STX)	PSP	[44,45,46]
*A. fundyense* Balech	Saxitoxins (GTX3, Neo, STX)	PSP	[47]
*A. hiranoi* Kita and Fukuyo	Goniodomin A	Cytotoxic, disturbs the actomyosin ATPase activity in diverse cell types	[48,49]
*A. insuetum* Balech	Not characterized	PSP	[50]
*A. leei* Balech	Not characterized, but neither PSTs nor spirolides	Mortality of finfish and rotifers	[51]
*A. minutum* Halim *	Saxitoxins (C1, C2, NEO, GTX1, GTX2, GTX3, GTX4)	PSP	[23,52]
*A. monilatum* (Howell) Balech	Goniodomin A	Highly toxic to fish and shellfish	[53,54]
*A. ostenfeldii* (Paulsen) Balech and Tangen	Saxitoxins (GTX3, GTX5); spirolides (A, B, C2, D2)	PSP (saxitoxins); toxicity to shellfish (spirolides)	[55,56,57]
*A. peruvianum* (Balech and Mendiola)	Hemolytic substances, Saxitoxins (B1, C1, C2, GTX2, GTX3, B1) Spirolides (C and D)	PSP	[58,59]
*A. pseudogonyaulax* (Biecheler) Horiguchi ex K. Yuki and Y. Fukuyo	Goniodomin A	Fish mortality	[60,61]
*A. tamarense* (Lebour) Balech *	Saxitoxins (C2, NEO, GTX3, GTX4)	PSP	[62,63]
*A. tamiyavanichii* Balech	Saxitoxins (C1, C2, GTX1, GTX2, GTX3, GTX5, STX)	PSP	[64]
*A. tamutum*	Saxitoxins (only genes found)	Antiproliferative activity on melanoma (A2058) and normal human lung fibroblast (MRC-5)	[26,32]
*A. taylori* Balech	Goniodomin A, Hemolytic substances	Effects on oyster larvae, sea-monkey mortality	[65,66]

PSP = paralytic shellfish poisoning; PSTs = paralytic shellfish toxins. * only some strains were found to be toxic.

**Table 2:** Bioactivities reported for *Alexandrium* species, mechanisms of action, cell lines or model organisms used. BIRC3 stand for Baculoviral IAP Repeat Containing 3; TRAF2 for TNF receptor-associated factor 2; DR3 for Death receptor 3; TNF for tumor necrosis factor; TNFS8 for TNF superfamily member 8; FASLG for Fas ligand; Gadd45 for Growth Arrest and DNA Damage; Foxl1 for forkhead box L1; Atg12 for autophagy-related protein 12/gene; ATP6V1G2 for ATPase H+ Transporting V1 Subunit G2; BNIP3 for BCL2/Adenovirus E1B19 kDa Interacting Protein 3; PINK1 for PTEN induced putative kinase1; NAA for N-acetyl aspartate.

Species	Sampling Location and Culture Conditions	Active Compounds/Extracts/Fractions	Bioactivities	In Vivo/In Vitro	Mechanism	References
*Alexandrium andersonii*	Mediterranean Sea [137]. -	Fraction B (digalactosyl diacyl glycerol and sulfoquinovosyl diacyl glycerol, were the predominant metabolites)	Cytotoxic (Anti-cancer)	In vitro investigation on: A549 cells	Down-regulation of anti-apoptotic molecules (BIRC3 and TRAF2) and up-regulation of death receptors (CD27, DR3, TNF, and TNFS8)	[133]
*Alexandrium andersonii*	Mediterranean Sea. -	Fraction D (rich in sterols and triacylglycerols)	Cytotoxic (Anti-cancer)	In vitro investigation on: HT29 cells	Induction of cell death with over expression of FASLG and TNF genes Up-regulation of Gadd45 alpha and gamma together with Foxl1 genes	[133]
*Alexandrium catenella*	Thau lagoon, France. Cultivated in L1 medium	-	Anti-viral	Herpes virus OsHV-1 μvar Oyster affected	Antagonist relationship that is established between the virus and *A. catenella*	[136]
*Alexandrium minutum*	Gulf of Naples. Cultivated in Keller medium	Fraction 3B (rich in carbohydrates (94%) and protein (4%))	Cytotoxic (Anti-cancer)	In vitro investigation on: PC-3, HT29, A549 cells	Up-regulation of autophagy-related protein Atg12 with a consequent increase in ATP6V1G2 and BNIP3 Up-regulation of mitophagy genes PINK1 and Parkin gene	[131]
*Alexandrium minutum*	Coastal marine waters of the NE Atlantic Ocean. Cultivated in F/2 medium, 27 of salinity (Guillard’s Marine Water Enrichment Solution) and Keller medium, 35 of salinity	*A. minutum* exudates	Anti-parasite	Anti-parasite activity against *Amoebophrya* sp.	Permeation of the membranes of *Amoebophrya* sp. dinospores, leading to the loss of dinofluorescence associated in turn with the loss of virulence of the parasite in culture	[135]
*Alexandrium ostenfeldii*	Coastal marine waters of the NE Atlantic Ocean. Cultivated in F/2 medium, 27 of salinity (Guillard’s Marine Water Enrichment Solution) and Keller medium, 35 of salinity	13-Desmethyl Spirolide C	Anti-Alzheimer	In vivo investigation in mice 3xTg-AD	Increase in NAA levels; Increase in synaptophytin and decrease in intracellular amyloid beta in the hypothalamus	[105]
*Alexandrium* sp.	-	Goniodomin A	Anti-angiogenic	-	Induction of morphological changes in human brain astrocytoma cells. Inhibition of ATPase activities of atrial myofibrils, natural actomyosin, and reconstitution of actomyosin.	[107,109]
*Alexandrium* sp.	-	Saxitoxin, neosaxitoxin, decarbamoyl saxitoxin, and tetrodotoxin	Rat sciatic nerve block (local anesthesia)	In vivo; evaluation of lethality, onset and duration of action for thermal analgesia (hot-plate testing), and motor block (weight-bearing)	Analgesia for 60 min with neosaxitoxin at 34 ± 2 micromol/L, saxitoxin at 58 ± 3 micromol/L, TTX at 92 ± 5 micromol/L, and decarbamoyl saxitoxin at 268 ± 8 micromol/L	[89]
*Alexandrium* sp.	-	Gonyautoxins 2/3 epimers	Treatment of Chronic headache	In vivo: investigation on 27 patients affected of chronic headache	Reversible block of the voltage-gated sodium channels at axonal level.	[91]
*Alexandrium* sp.	-	Tetrodoxins	Analgesic effects.	In vivo: subcutaneous injection of 30 µg.	Blockage of one subclass of sodium channels, NaV 1.7.	[130]
*Alexandrium tamarense*	Hiroshima Bay, Japan. Cultivated in ESM (Erd-Schreiber modified) medium pH8.2	Polysaccharide-based AT-toxin	Cytotoxic (Anti-cancer)	In vitro investigation on: HeLa, Vero, XC, CHO, and U937 cells	Induction of cell death in U937 by DNA fragmentation	[132]
*Alexandrium tamarense*	Cultivated in Keller medium	Total Extract	Anti-proliferative (Anti-cancer)	In vitro investigation on: MCF-7, MCF-10A, PC-3, and LNCaP cells	-	[134]
*Alexandrium tamarense*	Cultivated in Keller medium	Total Extract	Apoptotic (Anti-cancer)	In vitro investigation on: HepG2 cells.	-	[134]
*Alexandrium tamutum, A. andersonii, A. minutum*	Mediterranean Sea. Cultivated in Keller medium	Total Extract	Anti-proliferative (Anti-cancer)	In vitro on melanoma A2058 and MRC-5 cells	-	[32]

## Data Availability

Not applicable.
